# Spontaneous Discriminative Response to the Biological Motion Displays Involving a Walking Conspecific in Mice

**DOI:** 10.3389/fnbeh.2018.00263

**Published:** 2018-11-06

**Authors:** Takeshi Atsumi, Masakazu Ide, Makoto Wada

**Affiliations:** ^1^Department of Rehabilitation for Brain Functions, Research Institute of National Rehabilitation Center for Persons with Disabilities, Saitama, Japan; ^2^Japan Society for the Promotion of Science, Tokyo, Japan

**Keywords:** biological motion, social cognition, mice, comparative psychology, motion perception

## Abstract

Recent translational studies using mice have contributed toward elucidating the neural, genetic, and molecular basis of social communication deficits. Nevertheless, many components of visual processes underlying mice sociality remain unresolved, including perception of bodily-movement. Here, we aimed to reveal the visual sensitivity of mice to information on bodily motion using biological motion displays depicted by simple geometric dots. We introduced biological motions extracted from walking mice vs. corresponding meaningless scrambled motions, in which the spatial configurations of each path of dots were shuffled. The apparatus was a three-chambered box with an opening between the chambers, and each side chamber had a monitor. We measured the exploration time of mice within the apparatus during the test, with two types of displays being presented. Mice spent more time in the chamber with the scrambled motion displays, indicating that animals spontaneously discriminated stimuli, with the scrambled motion being relatively novel. Furthermore, mice might have detected socially familiar cues from the biological motion displays. Subsequent testing revealed that additional mice showed no bias to the static versions of the stimuli used in the Movie test. Thus, we confirmed that mice modulated their behavior by focusing on the motion information of the stimuli, rather than the spatial configurations of each dot. Our findings provide a new perspective on how visual processing contributes to underlying social behavior in mice, potentially facilitating future translational studies of social deficits with respect to genetic and neural bases.

## Introduction

Over the last three decades, studies have elucidated human social cognition based on visual cues produced by the eyes, face, and bodily-parts of other individuals, providing information about emotions and intentions (Happé et al., 2017). Animal studies develop our knowledge about the evolutionary and neural basis of cognition. Although rodents are one of the most accessible laboratory animals, knowledge about visual processing for social cues, including bodily-movement, remains limited.

The visual perception of motion related to social information is evolutionally fundamental (Troje, 2013). Researchers interested in the social significance of motion information have focused on assessing how biological motion is perceived (BM; Johansson, 1973). BM displays are usually made by attaching light sources on an actor's body and recording their movements in a dark environment. Motion is isolated from other sources of information, like shape and color; however, the displays are readily identified as depicting the actor's bodily movement and various actions (Troje, 2013). Some studies have shown that perception of BM is strongly linked to social cognition in humans (Pavlova, 2012). For instance, people with autism spectrum disorders (ASD), characterized by social deficits, are less sensitive to BM (Blake et al., 2003; Klin et al., 2009; Koldewyn et al., 2010).

Some studies of non-human animals have reported the ability for individuals to discriminate BM from comparative displays (cats: Blake, 1993; bottlenose dolphins: Herman et al., 1990). Recent studies, however, have distinguished between the ability to discriminate stimuli acquired through simple training and other discriminative responses (Pavlova, 2012). Some species cannot generalize their learning to novel BM displays (baboons; Parron et al., 2007; rats: MacKinnon et al., 2010; pigeons: Dittrich et al., 1998; Yamamoto et al., 2015; rhesus macaques: Vangeneugden et al., 2010). The discrimination training employed in these studies might induce a behavioral strategy in which the animals use local movements of dots as a discriminative cue.

In some cases, it seems reasonable to focus such natural behavioral repertoires on the perception of social signals. Studies of newly hatched domestic chicks employed imprinting techniques and showed their innate sensitivity to BM stimuli made from a video clip of a walking hen (Regolin et al., 2000; Vallortigara et al., 2005; Vallortigara and Regolin, 2006). A study of marmosets showed that they gaze longer at BM displays than other control stimuli, including static, inverted, scrambled, and rotated versions of the BM (Brown et al., 2010). Another study, focusing on the gazing behavior of dogs, examined the effect of oxytocin, which is a neuropeptide hormone implicated in reproductive and social behaviors (Kovács et al., 2016). The authors found that oxytocin enhanced differences in the gaze distribution of dogs between BM and control stimuli. A study of medaka fish showed that the BM stimuli induced shoaling behavior (Nakayasu and Watanabe, 2014). Further evidence of the social significance of BM was provided by a study with neural recordings of monkeys (Oram and Perrett, 1994). When viewing the BM display, the authors found that the anterior superior temporal polysensory (STP) area was activated, a region responsible for processing various socially relevant visual cues (Allison et al., 2000).

Previous studies suggested that mice might perceive visual cues, such as painful facial expressions, postures, or gestures (Langford et al., 2006, 2010), and whole-body actions in social contexts (Watanabe et al., 2016). Watanabe et al. (2016) demonstrated that videos of mouse social behavior are visually attractive to conspecifics, with animals staying longer in places with particular video clips. Two studies on rodents examined their acquired discrimination of BM displays by other species, such as a walking hen and human (MacKinnon et al., 2010; Foley et al., 2012). However, whether BM displays induce untrained behaviors remain untested. It remains unclear whether the visual preferences of mice are elicited by socially relevant motion information other than visual properties, such as shape and color.

This study aimed to elucidate the processing of motion information by mice using bodily actions as the core component of social cognition. Spontaneous discrimination of BM displays should be strictly linked to adaptive behavior. We examined whether mice differentiate BM displays of walking mice from control stimuli without any training (Movie test). The possibility that animals respond to the local motion of each geometric particle was examined by introducing scrambled motions as control stimuli originating from BMs, by altering their global appearance. In the subsequent tests, static versions of the BM displays were presented to examine how motion information contributes to behavioral modulation (Static image test).

## Materials and methods

### Subjects

Twenty-four male mice (*Mus musculus*) of C57BL/6N were purchased from Charles River Laboratories, Japan. Twelve mice were assigned to the movie test, and the remaining individuals were used in the static image test. The mice used in our study were 6~9 weeks old. The mice were housed in group cages, with four individuals per cage. The mice were provided with food and water *ad libitum*. The housing room was maintained at 23°C on a 12 h light/dark cycle. Before the study, the mice were not exposed to any of the experimental material in this study.

All animal procedures were performed in accordance with the institutional guideline for laboratory animals and approved by the animal care committee of Research Institute of National Rehabilitation Center for Persons with Disabilities (#28-AE1).

### Apparatus

We focused on whether the subjects showed spontaneous discriminative behaviors to the visual stimuli. We then developed an experimental set up based on a three-chambered sociability test apparatus for rodents (Moy et al., 2004). This is a standardized testing protocol used to examine the behavioral phenotype regarding the social communication capacity in a strain. In this protocol, the time spent in the chamber with a familiar or an unfamiliar conspecific of the subject is measured. Naïve and wild-type mice generally tend to stay longer in the chamber with any conspecific or with novel individuals of conspecific (Moy et al., 2004). Mice modulate such place preferences in the presence of real organisms and with the appearance of video recorded conspecifics (Watanabe et al., 2016). If mice are able to perceive BM, they would be expected to spend a different amount of time in the chamber with BM compared the chamber with the control stimuli, without any learning.

The testing apparatus was a rectangular, three-chambered box fabricated by O'Hara & Co., Japan. Each chamber was 20 cm L × 40 cm W × 22 cm H. Dividing walls were made from clear plexiglass, with small rectangular openings (5 cm W × 3 cm H), allowing access into each chamber. The chambers of the apparatus were cleaned using fresh paper chips with 70% ethanol before each trial. To present visual stimuli, a small LCD monitor (5 inch HDMI LCD (B), 800 × 480 resolution, cocopar) covered by a customized plastic case (5 cm L × 11 cm W × 15 cm H) was mounted on the wall on the opposite side of each door in both the left and right chambers. The top of the apparatus was an opening, above which a web camera (HD Webcam C615, Logicool) and a handy video camera (Everio GZ-E565, JVC) mounted above for the online measurements of trajectories of mice and for offline coding, respectively.

### Stimuli

**Figure 1:**
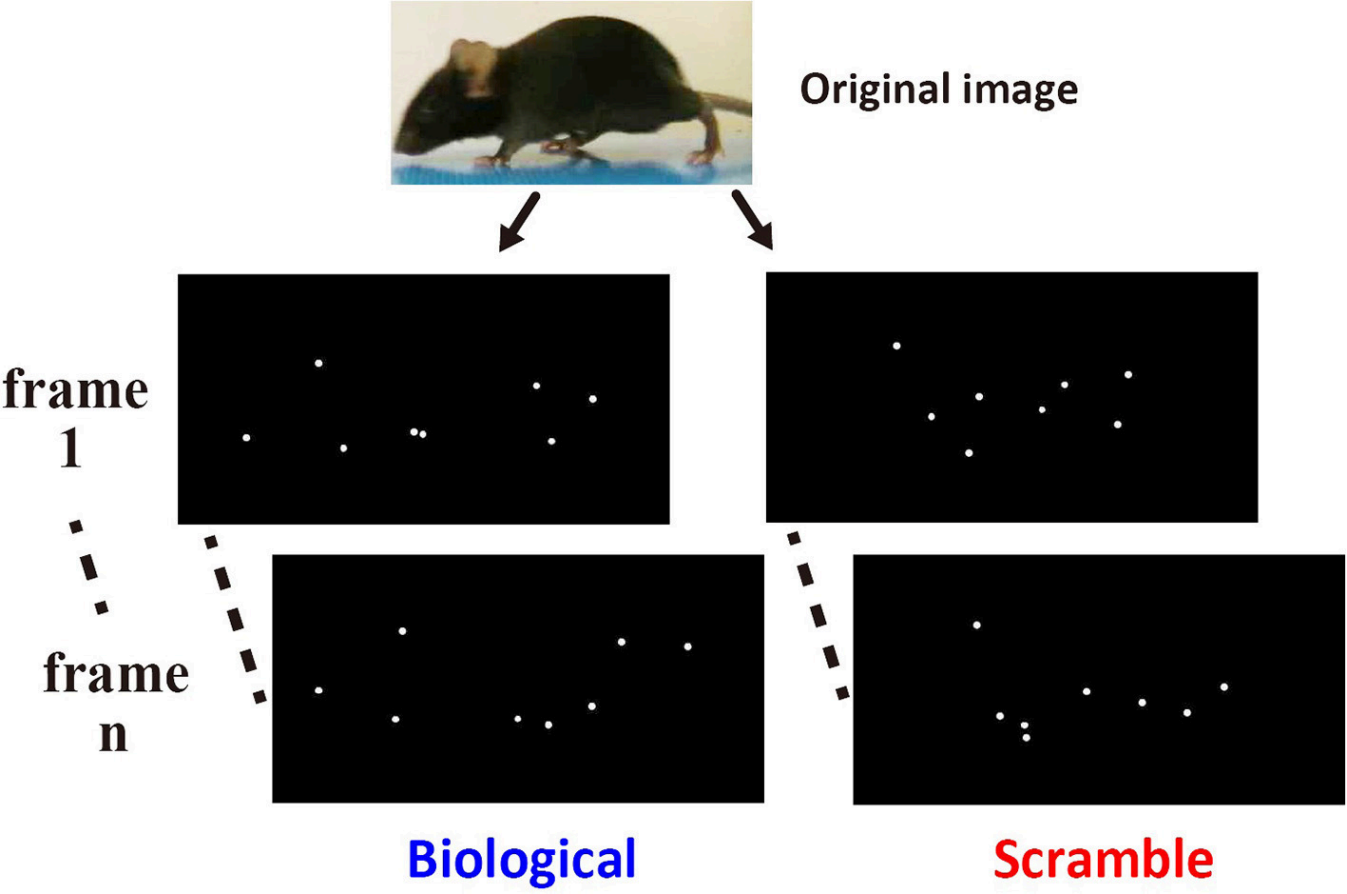
Example of the stimuli used in the Movie test. The left images show two non-successive frames from a point-light animation sequence depicting normal biological motion extracted from an original video clip (top). The right images show the corresponding frames from an animation containing the same dots undergoing the same local motions, only with their initial start locations shuffled.

The sensitivity of mice to BM stimuli was tested using a binary choice between a pair of point-light animations. The point-light animations were composed of 6~8 light points and were displayed at a speed of 24 frames/s. We created the stimuli employed in the current study using movie clips of real mice (Figure 1, top). We collected movie clips in which the adult male mice of C57BL/6N strain walk across the video camera's recording area. Scenes of mice walking were extracted from the sources, and involved at least one stroke of moving legs of the actor (14~20 frames). The end frame of the scene was manually defined by the experimenter. One stroke was defined as the period of time from the movement of the limbs of the mouse away from the floor in the first frame until the return of the limb to the same location. Each scene was looped during presentation by joining the last frame of the scene to the first frame. Three scenes by three different actors were obtained. We then created three stimulus sets for the Movie test involving both BM and control movie clips (Set 1~3), by using Adobe Flash CS6. White colored point-lights were placed at key points on the body area (tip of nose, root of an ear, hands of fore- and hind-limbs, root of the tail, and the midpoint of the tail) of the actor mouse in a scene, and dots occluded by a body part were not plotted on that particular frame. Then, the view of the original scene was replaced with a uniform dark background, with only the dots remaining. These modified movie clips from each scene were used as BM movies (Figure 1, left). To create control stimuli, the locations of each light-point at the beginning of the movie clip were shuffled, retaining entire motion path of each dot across the frames (scrambled motion: SM; Figure 1, right). For Static image test, the static stimuli were derived from the movie stimuli used in the Movie test. We extracted the first frames of each movie clip and introduced them as stimuli, and used three static pictures from the BM and SM clips, respectively. Presentation number and order of each stimulus was systematically controlled across the mice during experiments (see Procedure).

These stimuli were displayed using a self-made script in Matlab 2015a (Mathworks) with Psychtoolbox-3 (Brainard, 1997; Pelli, 1997) on MacBook Pro (Apple). Each stimulus was fitted within a 6.5 cm W × 4.5 cm H area on the monitor. The size of each dot on the monitors was 0.1 cm diameter. Examples of BM and SM movie clips are presented in the supplementary online material (Videos S1, S2, respectively).

### Procedure

**Figure 2:**
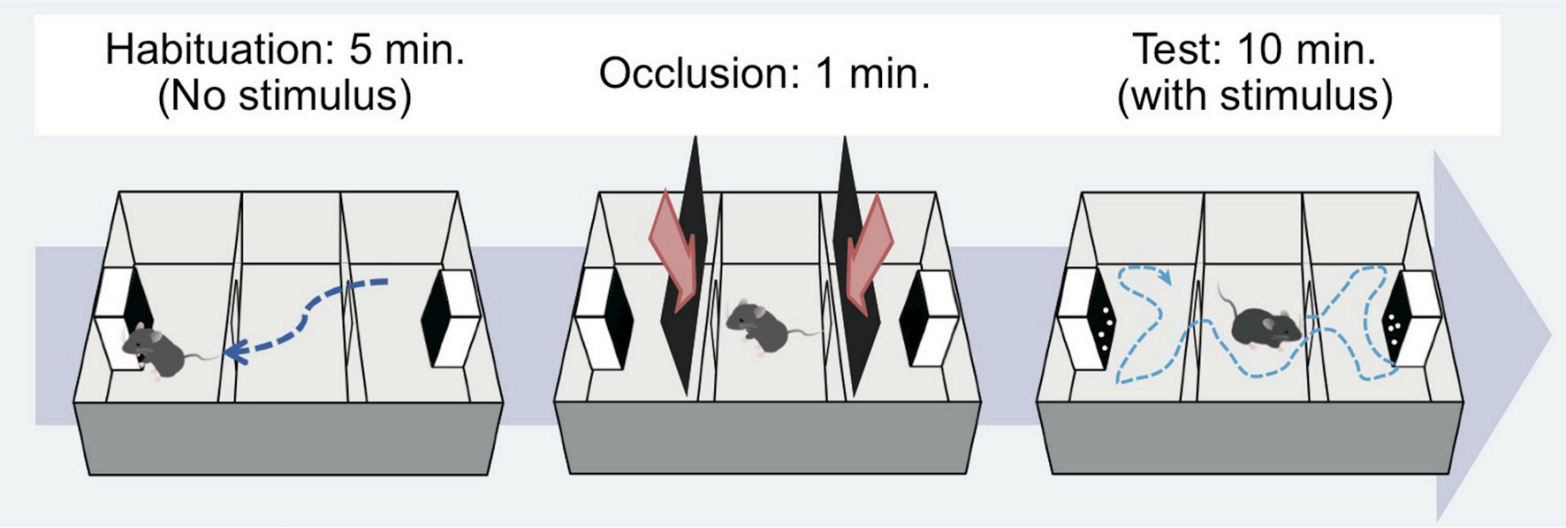
Apparatus and procedure. Mice were tested by using a three-chambered apparatus (Moy et al., 2004). Habituation phase (**left**): the subject moved around freely for 5 min to acclimate to the apparatus without any stimulus on the monitors. Occlusion phase (**middle**): the subject was placed in the central compartment for 1 min to prepare for subsequent testing. Test phase (**right**): the doors to the side compartments were opened and the mouse again freely moved for 10 min. Mouse movement during the experiments was recorded by web camera, and the time spent in each compartment was calculated using tracking software.

The test mouse was first introduced to the middle chamber. At the beginning of the habituation phase, the mouse was allowed to explore the entire test box for 5 min (Figure 2, left). The subject was returned to the center of the chamber at the end of this phase. Then, the doorways to the two side chambers were obstructed by opaque plastic occlusions for approximately 1 min (Figure 2, center). Then, the handy camera was turned on. Soon after that, a tracking software (ANY-maze version 4.75, Muromachi Kikai) and the presentation of the stimuli on each monitor were started, and the occlusions were immediately removed. One monitor presented the test stimulus, while the other showed the control stimulus, simultaneously. The subject was allowed to explore the entire test box for a 10 min session (Figure 2, right). During the 10 min period, one stimulus set of BM and SM was constantly presented. The amount of time spent in the left and right chambers during the period was estimated by the online tracking software and a human blind coder offline. An entry was defined as the center of the body area in one chamber. Each mouse experienced one trial of this testing per day, with a total of six trials throughout the experiment (over 6 days).

The location of the stimulus type (left vs. right side chamber) was systematically alternated between trials. The first chamber in which a BM movie was presented was counterbalanced across the subjects. Three stimulus sets were presented twice during the 6 days, and the chambers displaying each stimulus were alternated across days. Hence, the mouse was presented each movie clip once in each side chamber. We assumed that this manipulation would induce sufficient exploratory behavior by mice across days, ensuring no chance of acquiring any association between a specific stimulus and a stimulus type-dependent behavioral modulation (Simon et al., 1994; Rubinstein et al., 1997). The combination of the movie clips and the chambers where the stimuli were presented differed across days for each subject, and the order was counterbalanced across mice.

The procedure used in the Static image test was identical to that in the Movie test, except that mice were only tested for 2 days. The number of times that the static versions of each stimulus were presented was identical to those presented in the first 2 days of the Movie test.

## Results

### Movie test

**Figure 3:**
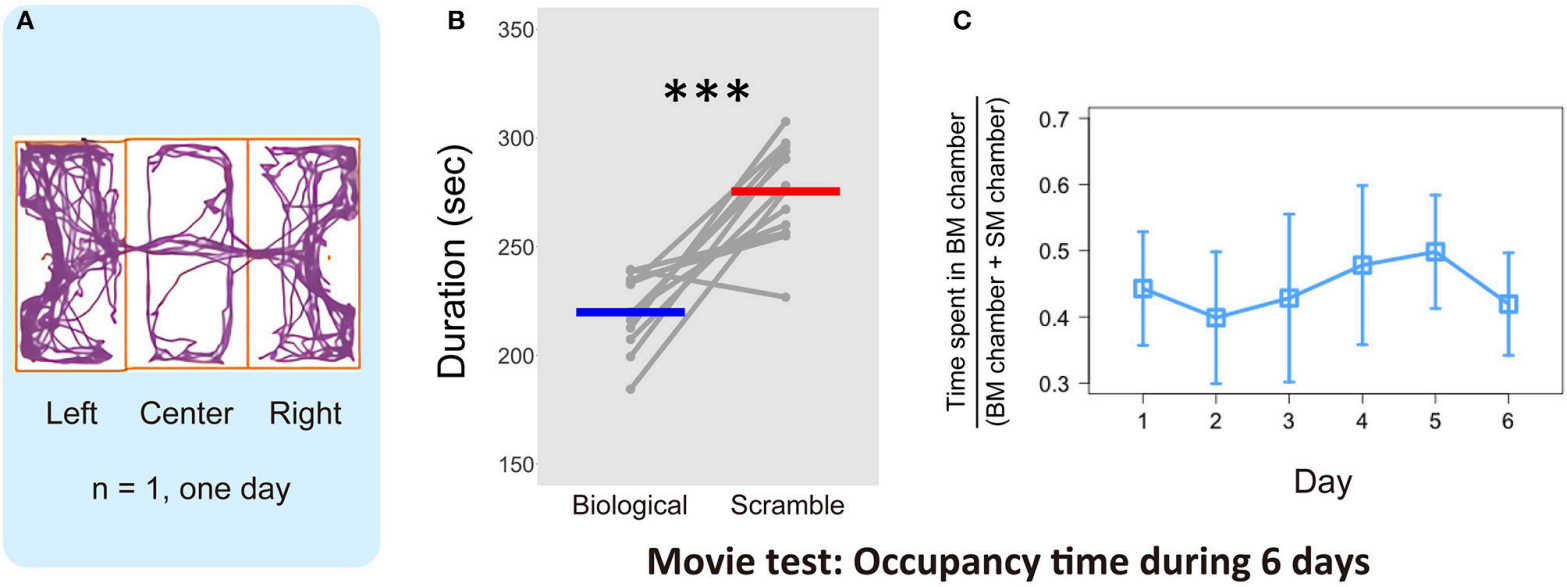
Results of the time spent in the chambers with each stimulus in the Movie test. **(A)** One representative example of mouse locomotion for 10 min in 1 day estimated by automated video tracking software. Purple line indicates the trajectory, and orange rectangles represent each chamber area. **(B)** Average time spent in each chamber over 6 days and individual plot of each mouse (*N* = 12). Gray dots and lines indicate the individual data, and horizontal bars colored blue and red show the average time in each condition, respectively. The difference of the average time between Biological and Scramble movies was significant (***p < 0.001). **(C)** Proportion of time spent in BM chamber across the 6 days (*N* = 12). Error bars represent the ±95% confidence interval of the mean.

**Figure 4:**
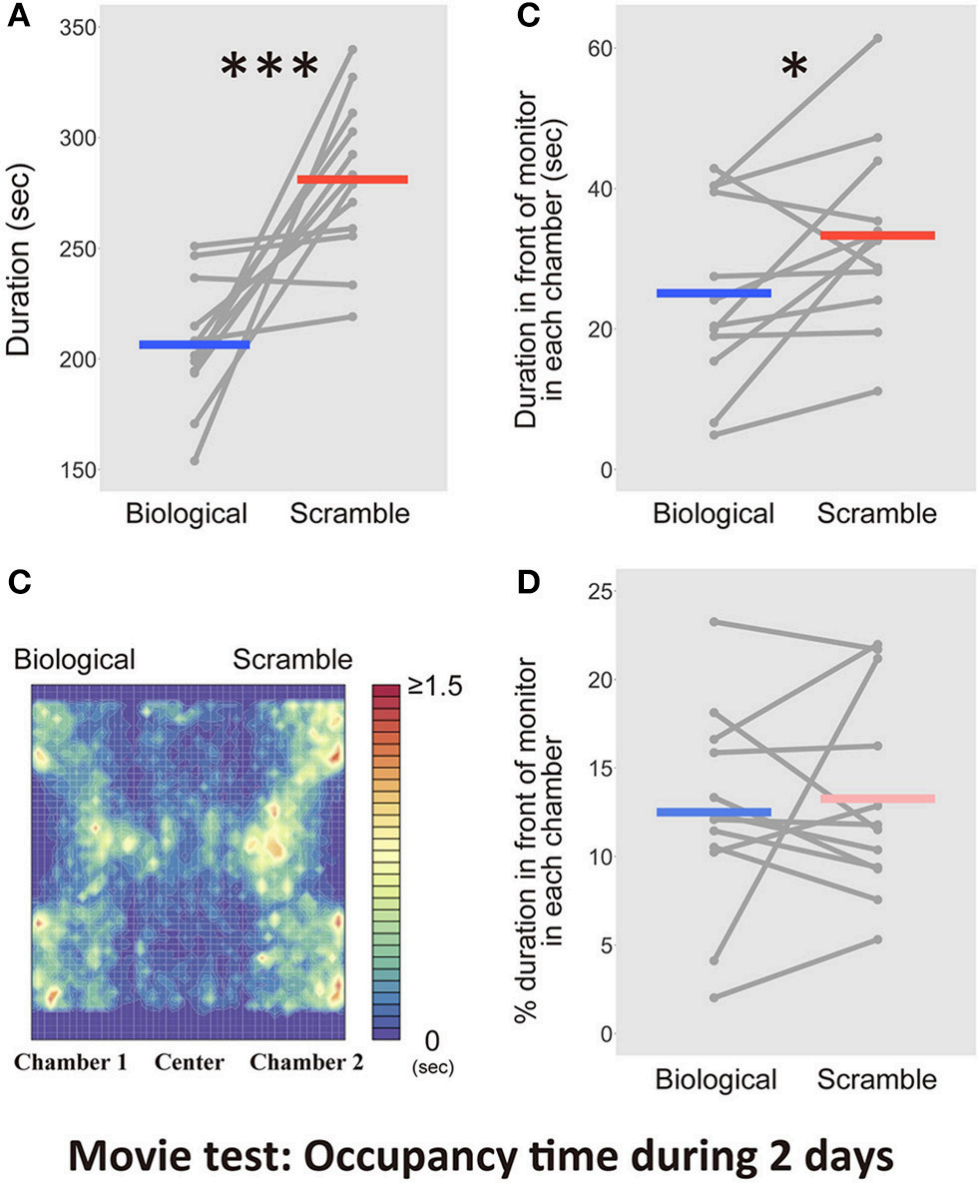
Results of the time spent in the chambers with each movie stimulus condition in the Movie test over 2 days (*N* = 12). **(A)** The difference between Biological and Scramble movies was significant (***p < 0.001). **(B)** A heat map of activity in the three-chambered apparatus during the test phases was estimated by averaging the occupancy times across the days for each mouse. **(C)** The time spent in front of the monitors. The difference between Biological and Scramble movies was significant (*p < 0.05). **(D)** Percentage of duration spent in front of the monitors during the entire time in each chamber. Gray dots and lines indicate the individual data, and horizontal colored bars show the averaged time in each condition, respectively.

Figure 3A shows one representative result derived using the tracking software. Over 10 min, the mice did not remain in a specific chamber, but moved in and out of the three chambers. We calculated the average time spent in each chamber with BM and SM stimuli across the days for each mouse (over 6 days, Figure 3B). We found that mice spent longer in the chamber with SM movies [paired *t*-test, *t*_(11)_ = −5.44, *p* = 0.0002, effect size: Cohen's *d* = 2.72, 95% confidence interval: CI = [−78.11, −33.11]]. This time difference between the stimulus conditions indicates that visual stimuli, even those depicted by simple moving dots, modulate mouse behavior in novel experimental apparatus. We confirmed the time-course effect to elucidate whether the subjects became habituated to the stimuli over the course of the experiment. The proportion of time spent in the BM chamber for the entire time spent in both chambers was calculated (6 days, Figure 3C). One-way repeated measure ANOVA revealed no main effect of days [*F*_(3.64, 40.07)_ = 1.58, *p* = 0.20, effect size: η${}_{\text{p}}^{2}$ = 0.13], with no indication of long-term habituation over time. The place preference in the chambers with SM stimuli was, therefore, maintained throughout the 6 days. In addition, the difference between conditions was already apparent in the first 2 days, in which each mouse experienced the presentation of each stimulus condition in both the left and right chambers [*t*_(11)_ = −4.42, *p* = 0.001, *d* = 2.29, CI = [−111.86, −37.54], Figure 4A]. Subsequent analysis for short-term habituation within a session revealed that the proportion of time spent in chambers with BM was stable for all minutes during the first 2 days [no main effect of time in each session: *F*_(__4.77, 567.74)_ = 0.77, *p* = 0.57, η${}_{\text{p}}^{2}$ = 0.006]. A paired *t* test showed a significant difference between two types of stimuli in the first minute of the 2 days [*t*_(11)_ = −3.20, *p* = 0.009, *d* = 1.65, CI = [−22.90, −4.23]]. The time differences between conditions that appeared in the early periods suggest that the behavioral bias of mice was not formed by any type of learning, rather their innate sensitivity to the stimuli.

Analyses for durations of residential behaviors demonstrated that the movie clips modulated the time mice spent in each chamber. This bias relied on the spontaneous responses of the animals and was strongly maintained throughout the experiment. Despite these clear differences, the occupancy time in the chamber provided insufficient information about whether the mice preferred to view the stimuli. Figure 4B shows a heat map that was estimated from the average occupancy time across the subjects. An example of the motion trajectory of a mouse (Figure 3A) and the heat map indicated that the subjects spent time in front of the monitors and in other areas, such as walls and corners. As a result, we questioned whether the effect of bias toward SM displays was true. Thus, we performed an additional analysis to estimate the occupancy time in front of each monitor. The visual acuity of mice is considered to be 0.5~0.55 cycles per degree (cpd) (Prusky et al., 2000; Prusky and Douglas, 2004). We used stimuli formed of 0.1 cm diameter dots. We ensured that each dot could be clearly viewed by mice from the midpoint of the chamber (5 cm away from the monitor, 0.88 cpd). We assumed that the distance was suited for the broad visual field of mice to view the global appearance of each stimulus (180° for monocular vision, and 40° for binocular vision; Dräger, 1978). The region of interest (within an ~5 cm diameter of the screens) and the occupancy time of these regions were estimated based on the frame-by-frame positions of mice coded by the software. We used the trajectories from the first 2 days and the average occupancy times across days for each subject in the statistical analysis. Figure 4C shows the time spent in front of the monitors with each condition. The occupancy time in front of the SM movie clip was longer than that in front of the BM clip [*t*_(11)_ = −2.33, *p* = 0.04, *d* = −0.62, CI = [−16.01, −0.44]]. Even in the area closer to the stimuli, mice remained longer in front of SM than BM displays.

The possibility that our animals avoided BM displays rather than preferred to approach SM stimuli remained. If the animals tended to keep a distance from BM clips, the time spent in front of the monitors with BMs would be relatively shorter than the time spent in front of the SM monitors. We analyzed the proportion of time spent in front of the monitors during the entire time in each chamber. There was, however, no difference between the conditions [*t*_(11)_ = −0.24, *p* = 0.82, *d* = −0.05, CI = [−2.68, 2.16]; Figure 4D]. Thus, the longer residency time in the SM chambers was associated with a visual preference for the control stimuli, rather than avoiding the BM chamber.

**Figure 5:**
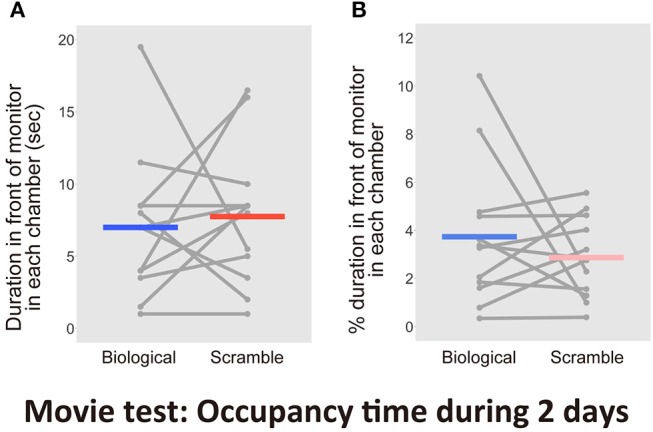
Results of the time spent in front of each monitor including attentional behaviors in the Movie test over 2days (*N* = 12). **(A)** The time spent in front of the monitors. **(B)** Percentage of duration spent in front of the monitors during the entire time in each chamber. Gray dots and lines indicate the individual data, and horizontal colored bars show the averaged time in each condition, respectively.

The location of mice was biased to the chamber with the SM display, but whether the animals paid attention to either display could not be determined. Previous studies reported attention-based behavioral repertoires toward biological motion stimuli in animals. Our tracking software was limited to detecting mice attentional behaviors only; thus, further analysis with a human blind-coder was required. First, the normal coding of the time spent in each chamber during the first 2 days performed by the human coder and the computer software were highly correlated, indicating significant reliability of human coding [*r* = 0.99, *t*_(46)_ = 43.09, *p* < 2.2 × 10^−16^, CI = [0.98, 0.99]]. Next, the time that the mouse spent within 1 cm diameter of the center of the LCD display's bottom, and paid attention to the monitor, was measured by the human coder. We measured the attentional behaviors including approaches into the region of interest and non-visual modalities, such as sniffing, touching, and nose poking. There was no difference between the time invested in attentional behaviors in front of the monitors during the entire time in each chamber [*t*_(11)_ = −0.38, *p* = 0.71, *d* = −0.15, CI = [−5.13, 3.63]; Figure 5A]. We also found no difference in the proportion of the time during the entire time in each chamber [*t*_(11)_ = 0.87, *p* = 0.40, *d* = 0.36, CI = [−1.32, 3.05]; Figure 5B]

**Figure 6:**
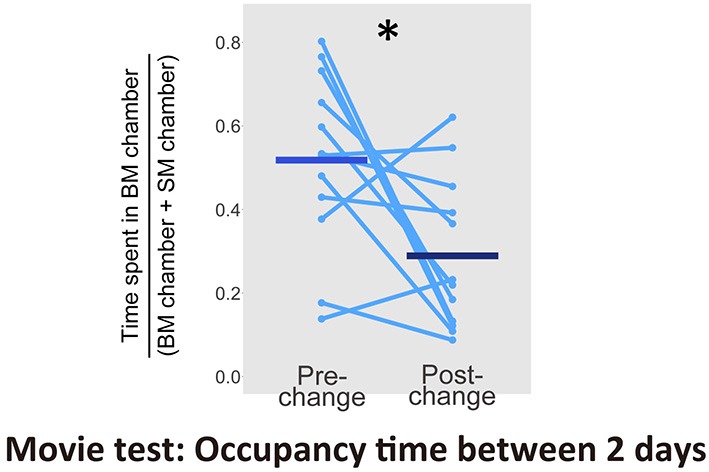
Proportion of time spent in front of monitors with BM chamber between pre- and post-change of stimulus sets in the Movie test (*N* = 12). Light blue dots and lines indicates the individual data, and horizontal bars show the averaged time. The difference between 2 days was significant (*p < 0.05).

Finally, we intended to determine the source of the modulation of mice residency time. An earlier study has already reported an orienting behavior toward non-socially relevant stimulus such as scrambled bot movements, and its visual novelty seemed to drive the behavior (Kovács et al., 2016). If the visual novelty of the display induced the orienting behavior of our mice, relatively greater preference for SM displays must be seen after movie clips were changed to new ones. We, therefore, calculated the ratio of time spent in front of displays (within about 5 cm diameter of the screens) with BM movies to total sum of the times spent in front of monitors with the 2 conditions for each subject. We then examined difference between the averaged times of the first pre- and post-change for stimulus set (Figure 6). The proportion of time spent with BM movies significantly decreased after the stimulus set change [a paired *t*-test, *t*_(11)_ = 2.66, *p* = 0.022, *d* = 1.15, CI = [0.04, 0.42]]. This indicates that the novelty of each set of stimuli influenced the residency time of the mice, and summing up, this novelty effect would be more obvious in SM condition.

### Static image test

**Figure 7:**
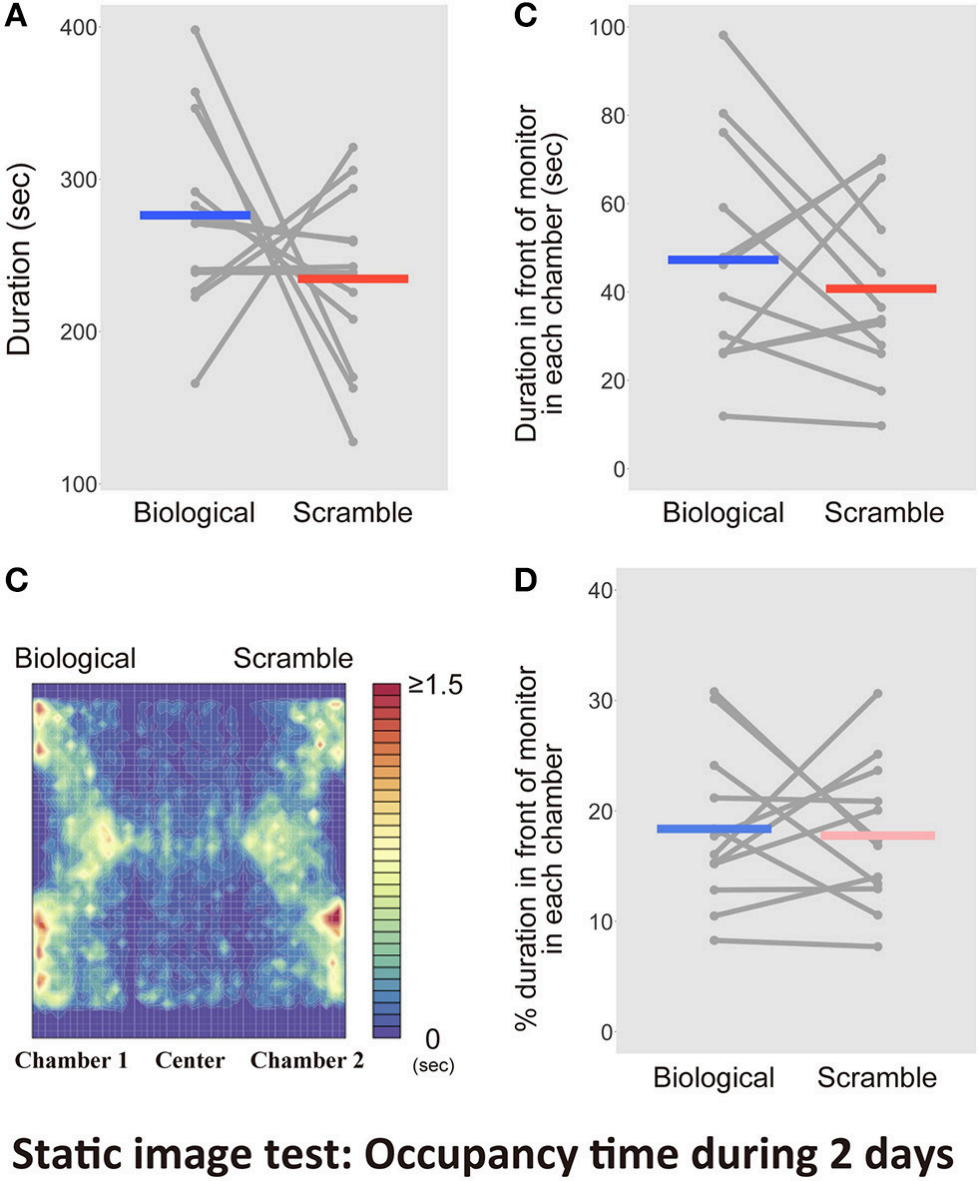
Results of the tests with the static picture stimuli in Static image test over 2 days (*N* = 12). **(A)** Bars indicate the time spent in the chambers with each type of static stimuli extracted from Biological and Scramble movies of mice. **(B)** A heat map of activity in the three-chambered apparatus during the test phases was estimated by averaging occupancy times across mice. **(C)** Time spent in front of the monitors estimated by the software. **(D)** Percentage of duration spent in front of the monitors during the entire time in each chamber. Gray dots and lines indicate the individual data, and horizontal colored bars show the averaged time in each condition, respectively.

The results of the Movie test demonstrated that the SM stimuli visually attracted mice, as they remained nearby for longer. Thus, the mice detected bodily-motion information of conspecifics from the BM displays, with the relative novelty of the SM displays potentially eliciting greater interest. However, it was still not known whether the motion information in the stimuli worked, or whether the simple spatial coordination of point-lights contributed in some way. The SM displays were obtained by rearranging the initial locations of each dot in the BM stimuli. If the special arrangements were relevant, static stimuli might also extract the same behavioral biases. Thus, we analyzed the responses of mice to static stimuli extracted from the movie clips employed in the previous test. We examined whether the discriminative behavior of mice relied on the motion information of the stimuli. We conducted the Static image test for 2 days, because the significant bias to SM stimuli was observed during the early period in the Movie test. Figure 7A shows the average time spent in each chamber with the static version of the BM and SM across the days for each mouse. We found no difference between stimulus condition [paired *t*-test, *t*_(11)_ = 1.19, *p* = 0.26, *d* = 0.67, CI = [−35.76, 119.23]]. Figure 7B presents the activity within the apparatus estimated from occupancy times. By analyzing the occupancy time in front of the images within 5 cm, we found no difference between the two conditions [*t*_(11)_ = 0.83, *p* = 0.43, *d* = 0.28, CI = [−10.94, 24.11], Figure 7C]. We analyzed the proportion of time spent in front of the monitors to the entire time in each chamber. There was also no difference between the two conditions [*t*_(11)_ = −0.30, *p* = 0.77, *d* = −0.11, CI = [−8.90, 6.79]; Figure 7D]. Thus, the behavioral bias obtained in the Movie test relied on the motion information of the stimuli. This result indicated that mice preferred the SM movie clips because of their integrated information, rather than local cues, such as the spatial configurations of dots.

**Figure 8:**
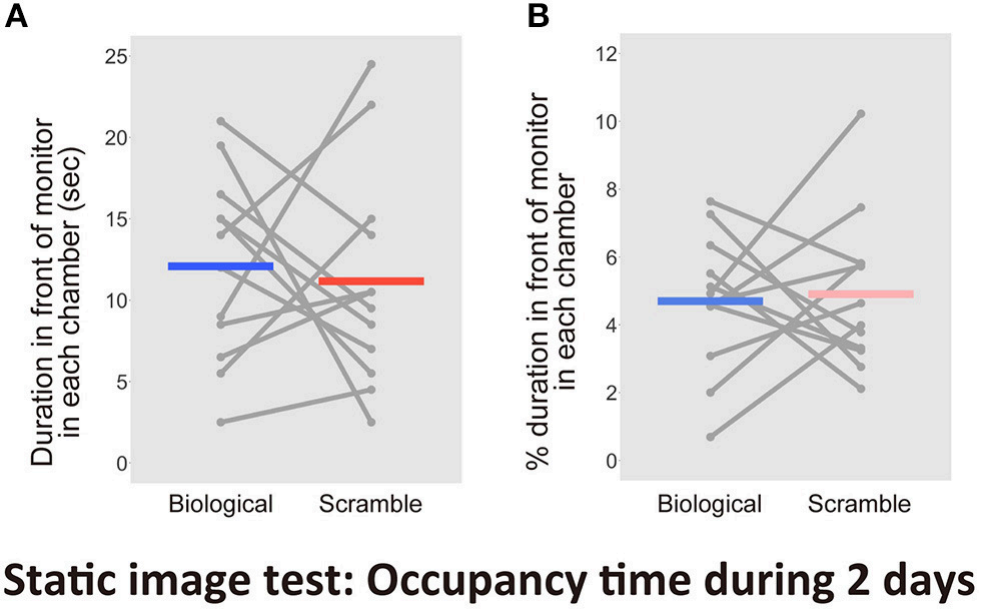
Results of the time spent in front of each monitor, including attentional behaviors, in the Static image test over 2 days (*N* = 12). **(A)** Time spent in front of the monitors. **(B)** Percentage of duration spent in front of the monitors during the entire time in each chamber. Gray dots and lines indicate the individual data, and horizontal colored bars show the averaged time in each condition.

Finally, we aimed to validate the time spent occupying the area within 1 cm of the monitors, which included the time spent for attentional behaviors. The entire time spent in each chamber during the first 2 days recorded by the human coder and the computer were highly correlated [*r* = 0.998, *t*_(46)_ = 102.87, *p* < 2.2 × 10^−16^, CI = [0.996, 0.999]]. The significant reliability of the human coding was again confirmed. The human coder then evaluated the amount of attention mice paid to the monitor. As a result, we found no difference between the time spent for attentional behaviors in front of the monitors [*t*_(11)_ = 0.34, *p* = 0.74, *d* = 0.15, CI = [−4.997, 6.830]; Figure 8A]. There was no difference between the proportion of residency time including attentional behaviors in front of the monitors in each chamber throughout the entire period [*t*_(11)_ = −0.23, *p* = 0.83, *d* = −0.10, CI = [−2.24, 1.82], Figure 8B].

## Discussion

The current study examined whether mice spontaneously discriminate BMs depicted by multiple point-light dots from a control stimulus. The Movie test revealed the animals have longer residency time in chambers with SM displays, in which the initial positions of the dots were shuffled but the original pendulum movement was preserved. This bias disappeared when we presented the static version of these stimuli. The results of the two consecutive tests suggested that mice are able to differentiate between the two types of stimuli by focusing on the entire appearance of the movies, rather than local features. Therefore, our results suggest that mice obtain bodily motion information, which is considered as a crucial source of social cognition (Blake and Shiffrar, 2007) from just the movie stimuli of point-light displays.

Previous reports argued that rodents discriminate BM displays from other stimuli; however, it is not known whether the animals perceived moving dots in an integrated way to perform tasks (MacKinnon et al., 2010; Foley et al., 2012). Non-human species often fail to group each motion of dots as a single stimulus (Dittrich et al., 1998; Yamamoto et al., 2015). Consequently, how local features contribute to the performance of the animal should be examined with care. The current study is the first to report that discriminative behavior in rodents is not elicited by the individual motion of dots nor their spatial configurations.

Discrimination between two types of stimuli was observed during exploratory behavior in Movie tests. Our data showed that exploration over three chambers was common among the mice. Hence, the mice had sufficient opportunity to view our stimuli during exploratory behaviors. This tendency was common across days, but the mice did not show any habituation for movie type-driven in both long-term and short-term periods. The behavior was not the result of avoiding BM displays. The bias toward SM movie clips seemed stable over 6 days period because of relatively greater visual interest in the stimuli than BM stimuli.

There could be some other possible reasons why we observed the bias toward SM, i.e., the bias was not a result of the on-display stimuli. One possibility is that an idiosyncratic movie clip attracted the mice and led to apparent longer residency time with SM. We thus, individually measured the residency times with each movie clip over 6 days (Supplementary Data). Comparisons between 2 stimulus types in each movie sets showed a trend in which the residency time with SM was longer than the other (Supplementary Figure 1). This suggests that the bias to SM displays was not the effect of a particular movie stimulus. Another concern is the possibility that our finding was simply obtained by chance. If a residency time in another area within the chambers, in which the monitors were not visible, such as corners, is more suited to explain the present result, it would be difficult to conclude that the visual perception really contributed. We checked the residential behavior of mice at corners of both sides of the chamber during the first 2 days. However, the bias toward SM was not due to the time spent at corners (see more details in Supplementary Data and Supplementary Figure 2).

Three-chambered test paradigms have been used in many translational studies on social deficits in mice, such as autism-spectrum disorders (ASD). Mice typically show preferences for socially novel conspecifics over familiar ones (Moy et al., 2004). The high reproducibility of these behavioral traits in mice, along with their preference for social novelty, has been repeatedly confirmed. This paradigm is broadly accepted by studies assessing the sociability of mice (Silverman et al., 2010). Considering the results of the current study, biological motion information extracted from the same species was more familiar to the subjects than scrambled motion information. Thus, the biased orienting behavior might be explained by a general preference for novel stimuli in mice. In actuality, an analysis focusing on the effect of changing the set of stimuli revealed that a visually novel set of movie clips modulated the residency time of the mice. Novelty preferences were also detected when examining BM perception in dogs (Kovács et al., 2016). Dogs received an intranasal administration of oxytocin, which is a neuropeptide hormone that is closely related to social cognition. The authors showed that the dogs exhibited longer fixation to scrambled motion displays than BM stimuli. They suggested that the ability of dogs to perceive BM more easily might enhance their visual attention to unfamiliar scrambled motions. The point-light displays employed in the current study were novel to the mice. Thus, our data support the concept that socially novel signals elicit visual preferences in mice. Further study is necessary to examine whether the familiarity-dependent orienting behavior in mice is innate, or whether it is formed by daily observations of other individuals.

Our results also indicated that the behavior of mice was not elicited by learning the point-light displays, because the bias appeared during the early period of the experiments. Spontaneous discrimination of biological motion stimuli suggests that individuals within a species use social information to communicate with other individuals. Biological motion stimuli often elicit orienting behaviors toward stimuli by humans or non-human animals. Such behaviors include approaching (Regolin et al., 2000; Vallortigara et al., 2005; Vallortigara and Regolin, 2006; Miura and Matsushima, 2016), shoaling (Nakayasu and Watanabe, 2014), and eye gazing (Simion et al., 2008; Kéri and Benedek, 2009; Klin et al., 2009). Sensitivity to body-movement contributes to non-verbal communications (Pavlova, 2012), which optimize adaptive behavior for processing signals produced by conspecifics. Our results confirm that mice also exhibit spontaneous discrimination of BM by conspecifics, supporting the concept that communication among animals depends on the appearances of bodily-movement. In the present study, our visual stimuli seemed insufficient to elicit any bias in attentional behaviors regarding non-visual modality (e.g., sniffing), but were sufficient for inducing approaches toward SM. This phenomenon might reflect modality-specific preferences to visual motion stimuli in mice.

The analysis of the reduced region of interest revealed that the occupancy time was significantly longer for a viewing distance within 5 cm (the midpoint of the left or right chamber) from the screen with SM. In the present study, it was still unclear whether the mice could distinguish each dot in our stimuli because of their poor visual acuity. Each dot presented on the monitor was 0.1 cm in diameter, which corresponded to 0.88 cpd when the mouse observed the stimuli at a distance of 5 cm from the monitor. While the visual acuity of mice is approximately 0.5 cpd (Prusky et al., 2000; Prusky and Douglas, 2004), it remains a possibility that the dots on the screen might be viewed as blurry by the animals. Each stimulus (consisting of 6~8 dots) was spread over nearly the entirety of the screen width of 6.5 cm, which was subtended at an angle of 66° on the mouse retina. Previous studies employing a similar visual angle of screen width reported neural activities of mice with a fixed view point, by presenting visual stimuli of lower spatial frequencies than that of the visual acuity of the mice (Niell and Stryker, 2008, 2010). In our study, the mice could freely move inside the apparatus and change their viewpoints and needed only to infer the global form, rather than explicitly distinguishing the individual dots. We therefore argue that our stimuli could elicit a behavioral bias toward unfamiliar, non-biological motion displays.

The results of our study do not enable us to determine whether the mice modulated their behaviors depending on physical and ecological contexts of BM. Our BM video clips presented walking from a fixed viewpoint, such as on a walker on treadmill. Although this type of movie allowed us to retain the whole-body appearance of the point-light walker, it might be physically unnatural for the mice. Many previous studies have shown that non-human animals could perceive BM displays of a fixed viewpoint (chicks: Regolin et al., 2000; Vallortigara et al., 2005; Vallortigara and Regolin, 2006, dogs: Kovács et al., 2016, primates: Oram and Perrett, 1994; Brown et al., 2010). These indicate that we could assume BM perception by non-human animals based on a treadmill-like walking action. Previous studies of rodents that reported the capacity of their discriminative learning revealed that the animals could differentiate these types of displays from the controls (MacKinnon et al., 2010; Foley et al., 2012).

Another concern is the possibility that our SM displays exhibited other actions of mice. It was difficult to regard the overall appearance of the SM displays as representing some other meaningful bodily actions, because we randomly rearranged the positions of each dot keeping the course of the motion trajectory. Thus, there was limited possibility that actions resembling those more commonly attractive than walking were randomly generated in these control movie clips. To elucidate whether our control stimuli were potentially meaningful, the relative attractiveness of various biological motions must be tested. To the best of our knowledge, it remains unknown whether non-human animals modulate their responses toward various types of BM depending on the bodily actions involved; addressing this point is a hurdle in the understanding of the context-dependent role of motion. To take this study forward, future work should explore whether context-dependent motion stimuli, such as physically and ecologically natural action repertoires of the mouse, induce different behavioral reactions in the animals.

What regions of the brain are involved in BM perception in mice? To date, various human studies have elucidated the key regions of the brain for perception. The posterior superior temporal sulcus (pSTS) was reported as the representative brain area and is thought to play an important role in social perception (Allison et al., 2000; Grossman et al., 2000; Grossman and Blake, 2001, 2002; Peuskens et al., 2005; Saygin, 2007). Some part of the fusiform gyrus and extrastriate cortex, which specifically respond to human bodily shapes, also seem to be responsible (Jokisch et al., 2005; Peelen et al., 2006). Furthermore, the premotor area responds to self-generated bodily movement, with observed bodily-motion produced by other individuals being related to the brain region (Saygin, 2007). It is difficult to identify most of the human brain regions that correspond to those in mice at present. However, some possible brain regions in mice might have similar perception to humans. A previous study reported mice and rats could detect the coherent motion of multiple point-light dots (Douglas et al., 2006). This global motion perception, like BM perception, depends on the spatiotemporal integration of moving dots in the visual system. Another study showed that viewing coherently moving dots induces the activation of both the frontal cortex and visual cortex in mice, whereas incoherent motion only activates the visual cortex (Han et al., 2017). The detection of unified motion involved in BM might be associated with this region. The mouse premotor area (M2) might share some homologous functions with humans for motor execution. M2 is thought to be crucial for goal-directed actions underlying motor planning (Gremel and Costa, 2013). Recent studies have elucidated the mice brain circuits with respect to social and emotional domains co-working with behavioral responses induced by visual cues from other organisms. The anterior cingulate cortex (ACC) and amygdala have been identified with the representative responsible brain regions to code affective or noxious signals from observed individuals (Jeon et al., 2010; Burkett et al., 2016). These brain activities also accompany the observation of itching behavior (Yu et al., 2017). Studies using mice have suggested that the brain circuit responsible for BM perception is found in the pathway from visual cortex through to the frontal cortex, M2, ACC, and amygdala. To examine this hypothesized circuit for the BM perception of mice, socially and emotionally valuable stimulus categories should be employed. A simple geometric moving visual stimulus simulating a potential predator induces the emotional behaviors of mice, such as freezing and flight responses (De Franceschi et al., 2016). Therefore, viewing the BM display made from potential predators might also activate the brain, including the ACC and amygdala. We should verify that the BM perception of mice reflects the visual system, which detects structural information from observed motion in the frontal cortex, and then engages it to motor planning in M2, and emotional information processing in the ACC and amygdala. Future studies targeting the brain circuits might reveal the convergence of perception of bodily motion derived from different brain structures acquired during the evolution of different species.

Our data suggested the innate sensitivity of mice to bodily motion information; however, this result must be carefully considered by examining whether we can extend this finding to social cognition. In humans, researchers have shown the linkages between BM detection and social skills by analyzing patients with social deficits (Blake and Shiffrar, 2007). A previous study demonstrated the impaired BM discrimination of rats with social deficits, such as an autism model (Foley et al., 2012). The researchers employed a discrimination learning procedure, not providing additional evidence of the impaired sensitivity toward BM. In other words, additional experiments are required to determine whether the behavioral bias toward SM displays is impaired in mice with social deficits, such as the ASD model.

Recent translational studies using mice have demonstrated the neural and genetic basis of social deficits, such as ASD (as reviewed by Provenzano et al., 2012). Mice are still useful for studies of its visual domain, because transgenic and knockout models of this disorder can be generated easily (Pinto and Enroth-Cugell, 2000). Studies using ASD model mice might be able to support the relationship between their responses to BM and other social behaviors. Aberrant sociality in mice is basically assessed by focusing orienting behaviors toward other individuals. Although a number of genetic mutations and interferences during early neurodevelopment induce ASD-like abnormal behaviors, it is not well known whether these alternations are associated with domain-general sociability or modality-dependent social behaviors. The results of this study provide a new approach toward elucidating the neural and genetic basis of the entire social behavior of mice from the complex background cognitive capacity.

## Conclusion

The mice modulated their orienting behavior depending on the BM displays showing a conspecific walking as depicted by simple geometric dots. There, however, remains a possibility that our control stimuli were not the best to test the perception of BM in mice. In this study, we examined their responses toward displays of walking-actions and of walking-actions with the initial dot positions rearranged. Future study should test the action-dependent attractiveness of BMs. To tackle the current topic is expected to improve our knowledge of socially relevant visual processing in the mammalian brain and might contribute to therapeutic screening for social communication deficits derived from altered visual recognition of bodily motion.

## Author contributions

TA, MI, and MW conceived and designed the experiments. TA, MI, and MW performed the experiments. TA analyzed the data and wrote the manuscript. All authors approved the final version and agreed to be accountable for all aspects of the work.

### Conflict of interest statement

The authors declare that the research was conducted in the absence of any commercial or financial relationships that could be construed as a potential conflict of interest.

## Supplementary material

Video S1 An example of Biological motion movie clip.

Video S2 An example of Scrambled motion movie clip.
