# Supplementary material for: Spontaneous Discriminative Response to the Biological Motion Displays Involving a Walking Conspecific in Mice
Source: Front Behav Neurosci. 2018 Nov 6;12:263. doi: 10.3389/fnbeh.2018.00263 (PMC6232871; doi:10.3389/fnbeh.2018.00263)
Supplement: Supplementary file 3 [file Data_Sheet_1.PDF]

Supplementary Material

Spontaneous discriminative response to the biological motion displays involving a walking conspecific in mice

Takeshi Atsumi*, Masakazu Ide, Makoto Wada*

*** Correspondence:**

Dr Takeshi Atsumi, Makoto Wada

atsumi-takeshi@rehab.go.jp, wada-makoto@rehab.go.jp

1 Supplementary Data

Analysis for individual movie clips. We addressed the possibility that the spontaneous discrimination between biological motion (BM) and scrambled motion (SM) movies occurred due to a specific stimulus. We individually measured the residency times with each movie clip (Set 1 ~ 3) over 6 days, in which all 12 mice experienced each set the same number of times. Supplementary Figure 1 shows the time spent in each movie clip, and we compared the stimulus combinations in each set separately. Comparisons between BM and SM condition in each movie sets showed a trend in which the residency time for SM was superior than BM, and paired *t* tests revealed the difference was statistically significant in 2 out of 3 sets (Set 1: $t_{11} = -3.17$, $p = 0.0089$, effect size: Cohen's $d = -1.67$, 95% confidence interval: CI = [-105.53, -19.09]; Set 2: $t_{11} = -3.28$, $p = 0.0074$, $d = -1.67$, CI = [-103.00, -20.22]; Set 3: $t_{11} = -1.78$, $p = 0.10$, $d = -0.97$, CI = [-95.93, 10.11]). This analysis suggests that it was hard to interpret our main finding, that is, the longer time spent in the chamber with Scramble motion displays was not a result of a particular movie stimuli.

Analysis for occupancy time at the corners. We checked the residential behavior of mouse at corners of the end of the both side chambers. We measured the total sum of time spent in front of monitors (within approximately 5 cm diameter of the screens) and at the corners in each subject during the first two days (Supplementary Figure 2). We, then, compared the averaged proportions of the time at the corners between the chambers with BM and SM movies. As a result, we found no difference between the conditions ($t_{11} = 0.069$, $p = 0.95$, $d = 0.01$, CI = [-0.04, 0.04]). This suggests that the bias towards Scramble motion was not due to the time spent at the corners.

2 Supplementary Figures

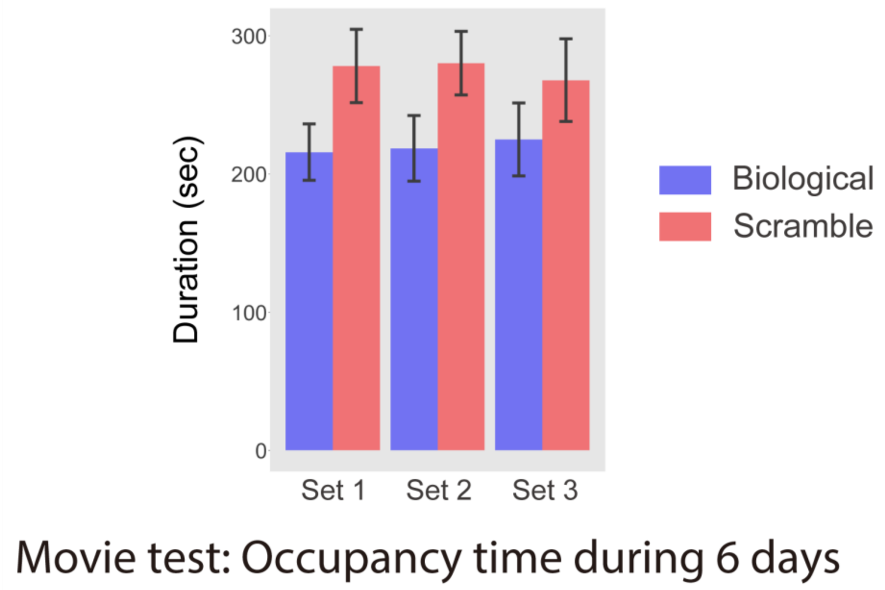

Supplementary Figure 1. Average time spent in each chamber with each movie clip over six days (N = 12). Error bars represent the ±95% confidence interval of the mean.

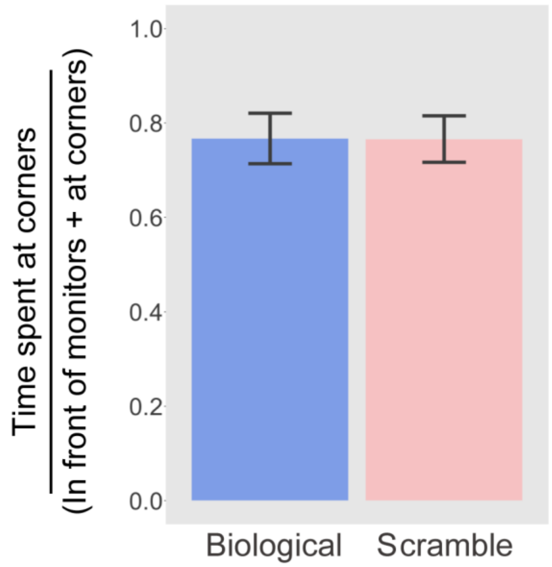

Movie test: Occupancy time during 2 days

Supplementary Figure 2. Proportion of the occupancy time at corners over two days (N = 12). Ratio between the time at the corners and the total sum of time spent in front of monitors and at the corners is indicated in each condition. Error bars represent the ±95% confidence interval of the mean.
